# Traumatic Brain Injury Broadly Affects GABAergic Signaling in Dentate Gyrus Granule Cells

**DOI:** 10.1523/ENEURO.0055-20.2021

**Published:** 2021-05-04

**Authors:** Alejandro Parga Becerra, Aric F. Logsdon, William A. Banks, Christopher B. Ransom

**Affiliations:** 1 Epilepsy Center of Excellence, VA Puget Sound Health Care System, Seattle, WA 98108; 2 Department of Neurology, University of Washington, Seattle, WA 98195; 3 Department of Physiology and Biophysics, University of Washington, Seattle, WA 98195; 4 Geriatric Research Education and Clinical Center, Veterans Affairs Puget Sound Health Care System, Seattle, WA 98108; 5 Division of Gerontology and Geriatric Medicine, Department of Medicine, University of Washington School of Medicine, Seattle, WA 98195

**Keywords:** epilepsy, GABAA receptor, GABAB receptor, hippocampus, TBI, tonic inhibition

## Abstract

Traumatic brain injury (TBI) causes cellular and molecular alterations that contribute to neuropsychiatric disease and epilepsy. GABAergic dysfunction figures prominently in the pathophysiology of TBI, yet the effects of TBI on tonic inhibition in hippocampus remain uncertain. We used a mouse model of severe TBI [controlled cortical impact (CCI)] to investigate GABAergic signaling in dentate gyrus granule cells (DGGCs). Basal tonic GABA currents were not affected by CCI. However, tonic currents induced by the δ subunit-selective GABA_A_ receptor agonist 4,5,6,7-tetrahydroisoxazolo[5,4-c]pyridin-3-ol (THIP; 10 μm) were reduced by 44% in DGGCs ipsilateral to CCI (CCI-ipsi), but not in contralateral DGGCs. Reduced THIP currents were apparent one week after injury and persisted up to 15 weeks. The frequency of spontaneous IPSCs (sIPSCs) was reduced in CCI-ipsi cells, but the amplitude and kinetics of sIPSCs were unaffected. Immunohistochemical analysis showed reduced expression of GABA_A_ receptor δ subunits and GABA_B_ receptor B2 subunits after CCI, by 43% and 40%, respectively. Activation of postsynaptic GABA_B_ receptors caused a twofold increase in tonic currents, and this effect was markedly attenuated in CCI-ipsi cells (92% reduction). GABA_B_ receptor-activated K^+^ currents in DGGCs were also significantly reduced in CCI-ipsi cells, confirming a functional deficit of GABA_B_ receptors after CCI. Results indicate broad disruption of GABAergic signaling in DGGCs after CCI, with deficits in both phasic and tonic inhibition and GABA_B_ receptor function. These changes are predicted to disrupt operation of hippocampal networks and contribute to sequelae of severe TBI, including epilepsy.

## Significance Statement

Traumatic brain injury (TBI) causes cellular and molecular changes that contribute to the clinical sequelae of TBI, including epilepsy, cognitive deficits, and mood disorders. Improved understanding of these cellular and molecular changes will inform therapeutic approaches to potentially disrupt epileptogenesis and treat symptoms. We investigated changes in GABAergic signaling of dentate gyrus granule cells (DGGCs) in a preclinical model of severe TBI [controlled cortical impact (CCI)] and identified early and persistent deficits in expression and function of both synaptic and extrasynaptic GABA_A_ receptors and GABA_B_ receptors. These broad changes in GABAergic signaling will alter function of DGGCs and are predicted to perturb hippocampal networks and contribute to epileptogenesis and cognitive deficits after TBI.

## Introduction

Traumatic brain injury (TBI) unleashes a cascade of cellular, molecular, and network changes that contribute to brain dysfunction after TBI, including cognitive deficits, mood/behavioral disorders, and seizures (Perry et al., 2016). Severe TBI is a potent risk factor for epilepsy, up to 50% of individuals with a penetrating head injury develop posttraumatic epilepsy (PTE; Annegers et al., 1998; Garga and Lowenstein, 2006). Dysfunction of signaling by the inhibitory neurotransmitter GABA is believed to importantly contribute to epileptogenesis after TBI (Hunt et al., 2013; Guerriero et al., 2015).

GABA acts via distinct receptor subtypes to influence essentially every physiological and pathophysiological process in the brain. GABA_A_ receptors containing γ subunits cluster at synaptic connections and are transiently activated by vesicular GABA release to cause synaptic or phasic inhibition. In many areas of brain, high-affinity extrasynaptic GABA_A_ receptors, containing either α5 or δ subunits, produce a tonic form of inhibition with distinct temporal and spatial characteristics compared with synaptic inhibition (Farrant and Nusser, 2005). In addition to activation by ambient GABA concentration, estimated to be in the range of 90–200 nm (Santhakumar et al., 2006), extrasynaptic GABA_A_ receptors are believed to undergo agonist-independent, spontaneous channel openings that support tonic inhibition (Belelli et al., 2009; Patel et al., 2016). GABA also activates GABA_B_ receptors, which are G-protein-coupled receptors (GPCRs) present on both presynaptic and postsynaptic cells (Padgett and Slesinger, 2010). GABA_B_ receptors produce intracellular signal transduction and multiple effects, including reduction of cAMP/PKA activity, presynaptic inhibition of vesicular GABA release (Otis et al., 1993), and direct activation of K^+^ channels (Isaacson et al., 1993).

Tonic inhibition is of particular interest in regard to TBI and PTE. Clinical experience and experimental data indicate that both excessive or deficient levels of tonic inhibition, caused by altered GABA transporter expression/function or fluctuating neurosteroid levels, can exacerbate seizures and epilepsy (Koepp et al., 2005; Maguire et al., 2005; Cope et al., 2009). Additionally, tonic inhibition is increased after experimental stroke and pharmacological or genetic correction of this increase is associated with improved motor recovery (Clarkson et al., 2010). These results highlight the importance of appropriate regulation of tonic inhibition for normal brain function and to mitigate pathology. Tonic inhibition is regulated over short time frames (seconds to minutes) by vesicular GABA release, dynamic GABA transporter function, voltage-dependent properties of GABA_A_ receptors, and the actions of GPCRs (Glykys and Mody, 2007; Ransom et al., 2010, 2013; Connelly et al., 2013a). GABA_B_ receptors potentiate tonic inhibition in several cell types, including dentate gyrus granule cells (DGGCs), cerebellar granule cells, interneurons, and thalamocortical neurons (Connelly et al., 2013b; Tao et al., 2013). This effect is mediated by postsynaptic GABA_B_ receptors and δ subunit-containing extrasynaptic GABA_A_ receptors and provides a link between GABAergic signaling at distinct receptor subtypes. In DGGCs, GABA_B_ receptors and δ subunit-containing extrasynaptic GABA_A_ receptors are both localized to perisynaptic/extrasynaptic portions of membranes (Kulik et al., 2003; Wei et al., 2003).

Published data supports GABAergic dysfunction after TBI. TBI causes a loss of GABAergic interneurons and reduced synaptic inhibition, which promotes hippocampal excitability (Huusko et al., 2015). Expression of many GABA_A_ and GABA_B_ receptor subunits in hippocampus are affected by TBI (mRNA and protein levels), including GABA_A_ receptor subunits believed to be involved in tonic inhibition (α4 and δ subunits; Kharlamov et al., 2011; Raible et al., 2012; Drexel et al., 2015). Tonic inhibition in DGGCs is also affected by experimental TBI but the reported results are variable with descriptions of enhanced, reduced, or unchanged tonic GABA currents after TBI (Mtchedlishvili et al., 2010; Pavlov et al., 2011; Gupta et al., 2012; Boychuk et al., 2016). This variability may relate in part to different TBI models, severity of injury, and timing and location of measurements after injury (i.e., ipsilateral vs contralateral hippocampus). Improved understanding of changes in GABAergic function after TBI is essential to critically assess mechanisms causing these changes and the role(s) of altered GABAergic signaling in epileptogenesis and the complex pathophysiology of TBI (Hunt et al., 2013; Glushakov et al., 2016).

We investigated the effects of controlled cortical impact (CCI) on GABAergic function of DGGCs in acute brain slices. Our results indicate early and persistent deficits in tonic inhibition after CCI, effects that were restricted to ipsilateral hippocampus. In addition, the function of synaptic GABA_A_ receptors and GABA_B_ receptors was reduced by CCI. These results indicate that GABAergic signaling in DGGCs is broadly affected by CCI. Altered function of GABA_A_ and GABA_B_ receptors are predicted to contribute to hyperexcitability of DGGCs/hippocampal networks and behavioral consequences of TBI.

## Materials and Methods

### Animals

Male C57 BL/6 mice were assigned to control or TBI groups. Electrophysiological and immunohistochemical assessments were performed at various time points after CCI; different groups of animals were used for experiments at 3 d, one week, two weeks, one month, and two months after injury. Animals were group housed and all protocols and procedures were approved by local IACUC.

### CCI

Mice aged five to six weeks were deeply anesthetized with inhaled isoflurane and placed in an electromagnetic cortical impactor system (ImpactOne device, Leica). Animal temperature was monitored and maintained during the procedure and post-surgery. A surgical window was opened over the scalp midline and a 3-mm craniotomy was performed over the right parietal cortex without disrupting the dura. CCI group received head trauma with impacts delivered over 50 ms with a 2-mm rounded metal tip at a velocity of 3.5 m/s to generate a 1-mm deformation in the cortical surface. After CCI, the injury was sealed with dental cement and animals observed until recovery. Sham group received craniotomy but did not receive impact. Animal weights, behavior, and wounds were followed daily for the following 3 d to evaluate recovery; all animals survived this procedure, a single animal required euthanasia because of wound dehiscence.

### Brain slice preparation

Animals were deeply anesthetized with 4% inhaled isoflurane then decapitated and brain dissected free from the skull. Brains were placed in ice-cold cutting solution with the following composition: 125 mm NaCl, 3 mm KCl, 26 mm NaHCo_3_, 1.2 mm NaH_2_PO_4_, 0.5 mm CaCl_2_, 4 mm MgCl_2_, 20 mm dextrose, and 1 mm kynurenic acid (osmolarity adjusted to 310 ± 5 mOsm). Solutions were continuously gassed with 95% O_2_/5% CO_2_. Transverse slices of hippocampus (300-μm thickness) were cut with a vibratome (Leica, VT1200) in ice-cold cutting solution and then incubated in cutting solution for at least 1 h at room temperature before experiments.

### Electrophysiology

Acute hippocampal brain slices were placed in a custom recording chamber with a volume of ∼3 ml and continuously superfused at a rate of 2–3 ml/min with a bath solution containing the following: 134 mm NaCl, 3 mm KCl, 26 mm NaHCO_3_, 1.4 mm NaH_2_PO_4_, 2 mm CaCl_2_, 2 mm MgCl_2_, 10 mm dextrose, and 1 mm kynurenic acid that was continuously gassed with 95% O_2_/5% CO_2_ to achieve a pH of 7.4. Bath solution osmolarity was adjusted to 295 ± 5 mOsm. DGGCs were visualized with an upright microscope using DIC/infrared optics (Slice Scope Pro2000, Scientifica). Whole-cell patch clamp techniques were used to digitally record membrane currents using a Multiclamp 700B amplifier, Axon Digidata 1550b A-D converter, and pClamp10 software (Molecular Devices). Data were recorded at 5–10 kHz and low-pass filtered at 1–2 kHz. Series resistance was monitored throughout experiments and compensated by 50%, recordings with a series resistance >20 MΩ or that had >25% change in series resistance were not considered for analysis. Micropipettes were produced from thin-walled borosilicate glass with filament using a micropipette puller (P-97 Sutter). Pipettes had resistances of 5–9 MΩ when filled with a solution designed to isolate GABA_A_ receptor currents that contained the following: 130 mm CsCl, 10 mm EGTA, 10 mm HEPES, 10 mm QX-314 chloride, 4 mm NaCl, 0.5 mm Na_2_GTP, and 4 mm Mg-ATP, with adjusted pH 7.25 with CsOH. Osmolarity was adjusted to 280 ± 5 mOsm. When assessing K^+^ currents a K-gluconate internal solution was used, containing the following: 125 mm K-gluconate, 10 mm KCl, 1 mm EGTA, 10 mm HEPES, 4 mm NaCl, 0.5 mm Na_2_GTP, and 4 mm Mg-ATP, with adjusted pH 7.25 with KOH. Kynurenic acid (1 mm) was included in bath solution to block ionotropic glutamate receptors for all patch clamp experiments. Membrane currents were measured at −70 or −110 mV (for K^+^ currents). All experiments were performed at 32–34°C.

### Drug administration

Tonic GABA currents were measured by focal application of the GABA_A_ receptor antagonist bicuculline methiodide (40 μm). Focal application was made by pressure ejection (Picospritzer III, Parker Hannifin) from a micropipette positioned above the tissue surface ∼50 μm from the recorded cell and triggered by acquisition software, typically with 2-s duration pulses delivered every 2 min (5–10 psi). Effects from the small volume of bicuculline released during focal pressure ejection resolved within 60 s. Other drugs were bath applied via bulk bath solution. Activation of GABA_A_ receptors containing δ subunits was accomplished by application of the agonist 4,5,6,7-tetrahydroisoxazolo[5,4-c]pyridin-3-ol (THIP) at the following concentrations 0.3, 1, 3, 10, and 30 μm. GABA_B_ induced tonic current changes were assessed by application of GABA_B_ receptor agonist baclofen (10 μm) and GABA_B_ receptor antagonist CGP55845 (CGP; 10 μm). When assessing GABA_B_ receptor effect through G_i/o_, guanosine 5’-O-(2-thiodiphosphate) (GDP-β-s; 0.5 mm) was used in the intracellular solution. Baclofen (100 μm) was focally-applied via pressure ejection for measurements of GABA_B_ receptor activated K^+^ currents. Chemicals were purchased from Sigma and Tocris.

### Immunohistochemistry (IHC)

Expression levels of GABA_A_ receptor δ subunits and GABA_B_ receptor B2 subunits were assessed by IHC (Schneider Gasser et al., 2006). For immunohistochemical experiments, brains were fixed via cardiac perfusion (perfused with 20-ml PBS with 1000 units heparin and then 35- to 50-ml paraformaldehyde). Brains were fixed overnight in 10% formalin and then stored in 30% sucrose at 4°C until ready to cryosection. Brain slices (50 μm thick) were cut with a cryostat and conditioned with 1 m PBS (4 × 20 min). Tissue was permeabilized by incubating in PBS containing 0.25% Triton X-100 and 0.01% sodium acetate (1 h) and then rinsed with PBS containing 0.1% Triton X-100 (PBST; 3 × 5 min). Antigen retrieval for GABA_A_ receptor δ subunits, NEUN, and GFAP was accomplished in PBS with 10 mm sodium citrate titrated to pH 6 at 80°C (1 × 30 min) and slices were stored in blocking buffer overnight at 4°C (PBS containing 2% BSA, 2% donkey serum, 0.25% Triton X-100). Antigen retrieval for GABA_B_ receptor B2 subunits was accomplished in PBS with 0.05% Triton X-100 at 50°C (30 min) and then slices stored in blocking buffer (same as above but with 0.25% Triton X-100). Slices were incubated with primary antibodies overnight at 4°C [anti-GABA_A_R δ Rb polyclonal diluted 1:3000 (R&D Systems, catalog #PPS090B); anti-GABA_B_R β_2_ goat polyclonal diluted to 5 μl/ml (R&D Systems, catalog #AF1188); anti-NEUN rabbit polyclonal diluted 1:2000 (Abcam, catalog #ab104225); anti-GFAP chicken polyclonal diluted 1:2000 (Millipore, catalog #AB5541)]. Primary antibody was washed with PBS containing 1% BSA, 0.25% Triton X-100 (4 × 20 min). Slices were incubated with secondary antibodies at room temperature for 2 h [Cy3 donkey anti-rabbit IgG (Jackson ImmunoResearch, catalog #711-165-152) diluted 1:500 or Alexa Fluor 488 donkey anti-goat or anti-chicken IgG diluted 1:2000 (Jackson ImmunoResearch, catalog #705-545-147 and catalog #703-545-155, respectively)]. Tissue was reconditioned by washing with PBST (3 × 5 min) before staining with Fluoromount-G with DAPI (Invitrogen) and mounting on glass slides. Brain slices were imaged using an upright microscope (Eclipse Ni-E, Nikon). Tiled fluorescent images of the hippocampus were acquired using NIS-Elements software at 40× magnification using 390-nm light for DAPI, 488 nm for Alexa Fluor and 560-nm light for Cy3.

### Analysis

Electrophysiological recordings were analyzed with Clampfit (pClamp v10.6) and Origin (Microcal) software. Tonic GABA currents were measured as the change in mean holding current induced by the GABA_A_ receptor antagonist bicuculline. Histograms of current were constructed at baseline and at peak of bicuculline response and fit with a Gaussian function using Levenberg-Marquardt algorithm provided in Clampfit; the central moment of the Gaussian distribution defined mean holding current. Dose–response data were fit with a Hill equation of the form:

$$\text{I }={\text{ I}}_{\text{max}}*\;{\left[\text{THIP}\right]}^{\text{n}}/{\text{ EC}}_{\text{5}0}{}^{\text{n}}*\;{\left[\text{THIP}\right]}^{\text{n}}$$,

where I_max_ is peak current, EC_50_ is half-maximal effective concentration of THIP, and n is Hill coefficient. Spontaneous IPSCs (sIPSCs) were detected as negative-going events and sIPSC parameters (frequency, amplitude, rise/decay times) were quantified using a template matching function in Clampfit. Immunohistochemical images were analyzed using ImageJ (v1.48d). Dentate gyrus granule cell layer (GCL), molecular layer (ML), and hilus were delineated on a DAPI-stained, paired image, and set as region of interest (ROI). The mean gray scale value of ROI was determined from individual sections (background-subtracted) and recorded as the intensity of antibody staining for GABA_A_ receptor δ subunits and GABA_B_ receptor B2 subunits. Group data are presented as mean and SEM, and statistical comparisons were made using either a one-way or two-way ANOVA and two-tailed, unpaired *t* test included in software packages (Origin, Microsoft Excel), with a *p* < 0.05 considered significant.

## Results

### Histologic effects of CCI model

**Figure 1.**
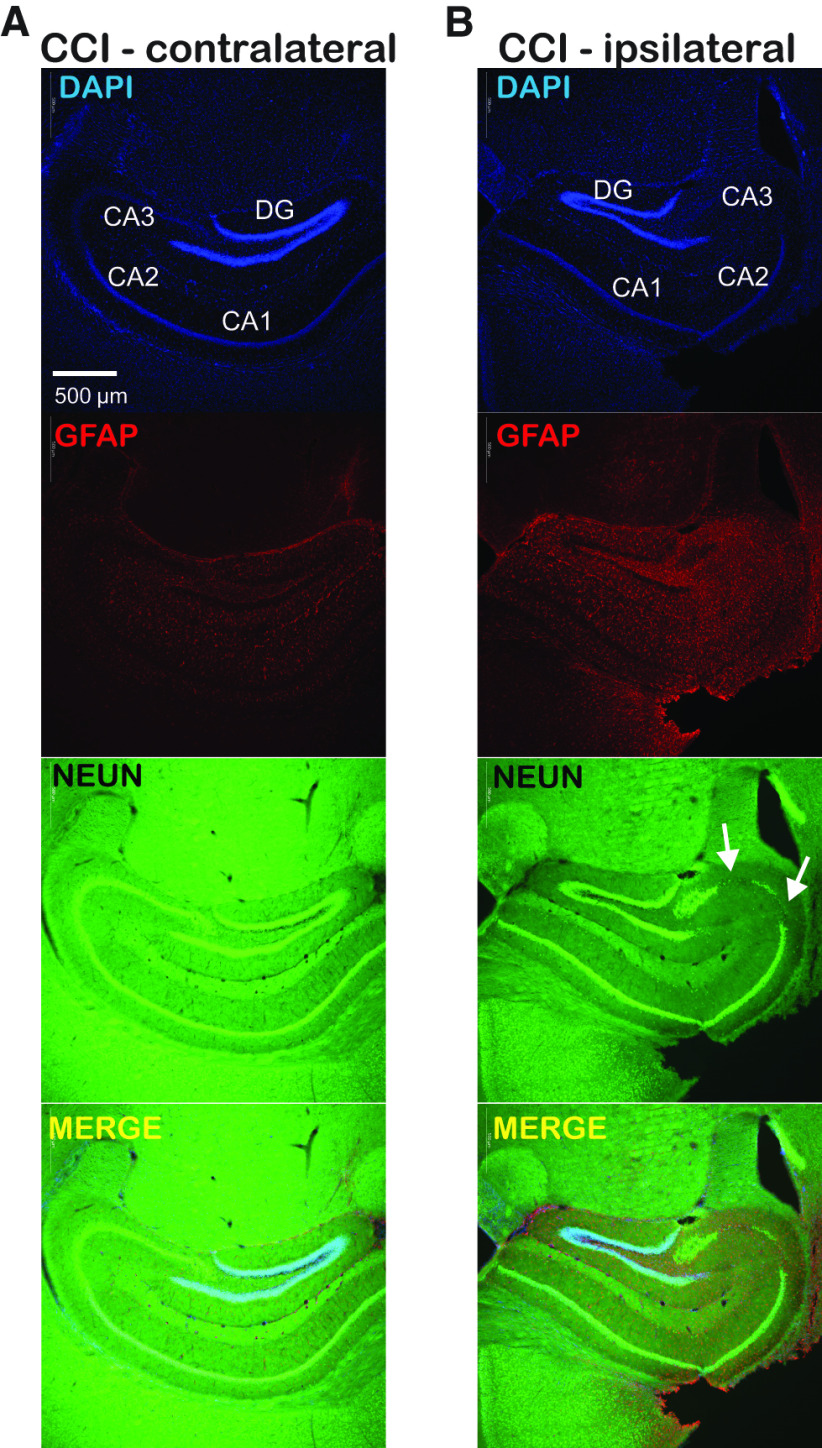
CCI produces persistent astrogliosis and loss of CA3 neurons that are restricted to the ipsilateral hippocampus. ***A***, Immunofluorescent images of contralateral hippocampus one month after CCI with DAPI (blue; nuclear), GFAP (red; astrocyte), NEUN (green; neuron), and merged images. Hippocampal subregions are labeled within the DAPI panel. ***B***, Immunofluorescent images from ipsilateral hippocampus after CCI showing DAPI, GFAP, NEUN, and merged images as in ***A***. Persistent astrogliosis after CCI is seen as increased GFAP staining, arrows indicate region of neuronal loss in the CA3 region within the NEUN panel. The astrogliosis and loss of CA3 neurons seen in ipsilateral hippocampus one month after CCI is absent in in the contralateral hippocampus; there is relative preservation of DGGCs targeted for our recordings, indicating these cells remain viable after CCI. Scale bar (500 μm) in upper left panel applies to all images.

CCI creates a direct and visible injury to the cortical surface but our focus is on CCI-related changes in the hippocampus, which is not directly affected by CCI. To assess the effects of our CCI model on neuronal survival and neuroinflammation/astrogliosis in the hippocampus we performed immunohistochemical staining for the neuronal marker NEUN and GFAP at four weeks after injury (Fig. 1). Qualitative assessment of these immunohistochemical images indicate preservation of neurons targeted for whole-cell recordings in dentate gyrus, in hippocampus both contralateral and ipsilateral to CCI. We did not quantify NEUN+ cell numbers but loss of neurons in area CA3 was visibly-appreciable in ipsilateral hippocampus in all slices examined from CCI-treated animals (*n* = 4 animals), this loss of CA3 neurons was not apparent in hippocampus contralateral to CCI (Fig. 1). GFAP staining was increased four weeks after CCI in the ipsilateral hippocampus compared with contralateral hippocampus. These results validate histologic alterations of hippocampus four weeks after CCI and indicate a strong laterality to these changes with evidence of persistent astrogliosis, a marker of neural injury and neuroinflammation (Colombo and Farina, 2016).

### CCI effects on tonic GABA currents in DGGCs

**Figure 2.**
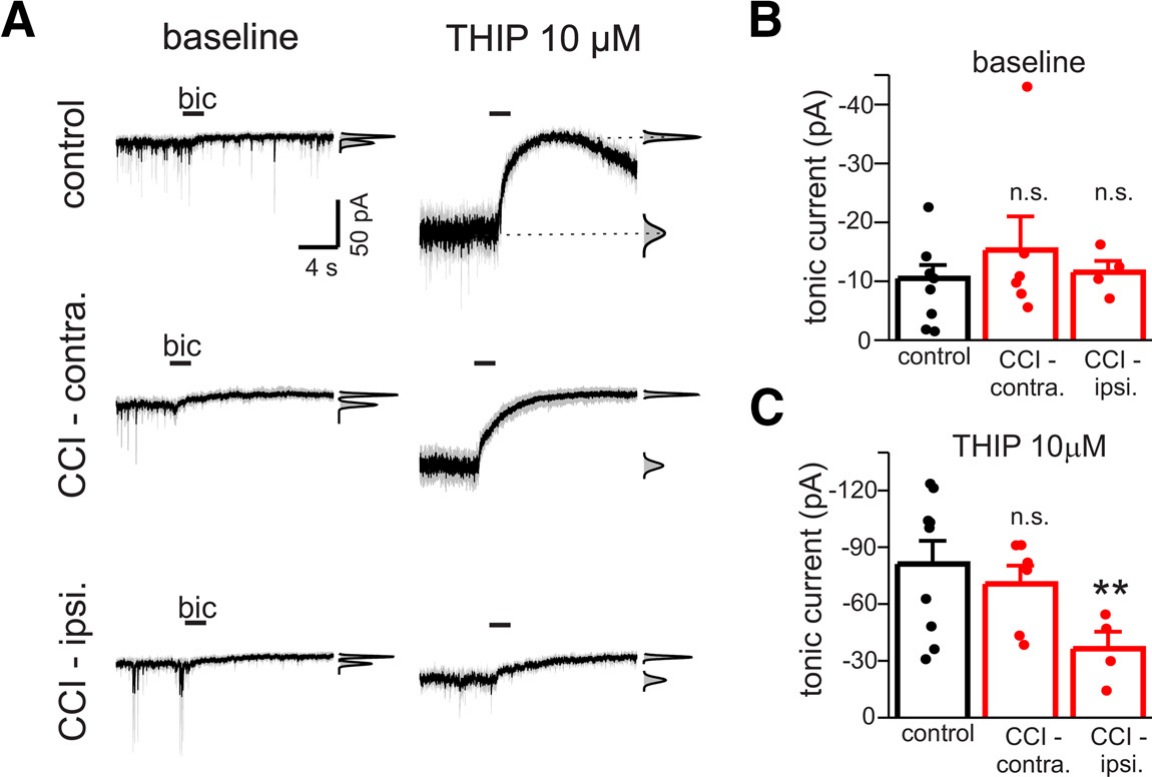
CCI reduces THIP-induced tonic currents, but not basal tonic currents, in ipsilateral DGGCs. ***A***, Membrane currents of DGGCs in response to focal application of the GABA_A_ receptor antagonist bicuculline (Bic) application before and during exposure to THIP (10 μm). CCI data recorded two weeks after injury. Illustrated currents are mean response (black line) of three focal applications of Bic overlaid with the individual responses to Bic in each cell (gray line). Insets to right of each trace represent all-points histogram and Gaussian fits used to measure mean holding current before and after Bic application; tonic current amplitude is defined as difference in mean holding current produced by Bic. Horizontal bars represent period of drug application in this and subsequent figures. Holding potential was −70 mV. ***B***, Mean baseline tonic current (±SEM) of DGGCs in control, CCI-contra, or CCI-ipsi slices. Baseline tonic currents were unaffected by CCI two weeks after injury. Solid circles represent data from individual cells in this and subsequent figures. ***C***, Mean THIP-induced tonic current of DGGCs in control, CCI-contra, or CCI-ipsi slices. CCI significantly reduced amplitude of THIP-induced currents in CCI-ipsi cells only; **p < 0.01, n.s., not significant.

We measured tonic GABA currents in DGGCs of acute hippocampal brain slices from control/sham-operated animals and animals exposed to CCI. Recording conditions were designed to isolate GABA_A_ receptor currents (CsCl pipette solution with QX-314, extracellular kynurenic acid to block glutamate receptors). Tonic GABA currents were measured as the change in mean holding current at −70 mV produced by focal pressure ejection of the GABA_A_ receptor antagonist bicuculline (40 μm) from a micropipette positioned 30–50 μm from DGGC soma; mean holding current was determined as the center of Gaussian function fit to histograms of current data (Fig. 2*A*). Initial experiments were done on animals two weeks after CCI (i.e., 34–39 d old). Baseline tonic currents were similar between DGGCs from control animals and in DGGCs from both the contralateral and ipsilateral hippocampus of CCI-treated animals (one-way ANOVA, *p* = 0.89; *n* = 4–8 cells; Fig. 2*A*,*B*). However, significant differences in tonic currents were observed in the presence of the GABA_A_ receptor δ subunit-selective agonist THIP (10 μm; one-way ANOVA, *p* < 0.01; Meera et al., 2011). CCI reduced THIP-induced tonic currents in ipsilateral hippocampus by 55% compared with control cells (control: −81.6 ± 12.2 pA vs CCI-ipsi: −36.4 ± 9.0 pA, *n* = 4–8 cells, *p* < 0.01; Fig. 2*A*,*B*). THIP-induced tonic currents were unaffected in contralateral hippocampus (CCI-contra: −70.6 ± 9.6 pA, *n* = 6 cells, *p* = 0.55; Fig. 2*A*,*C*).

**Figure 3.**
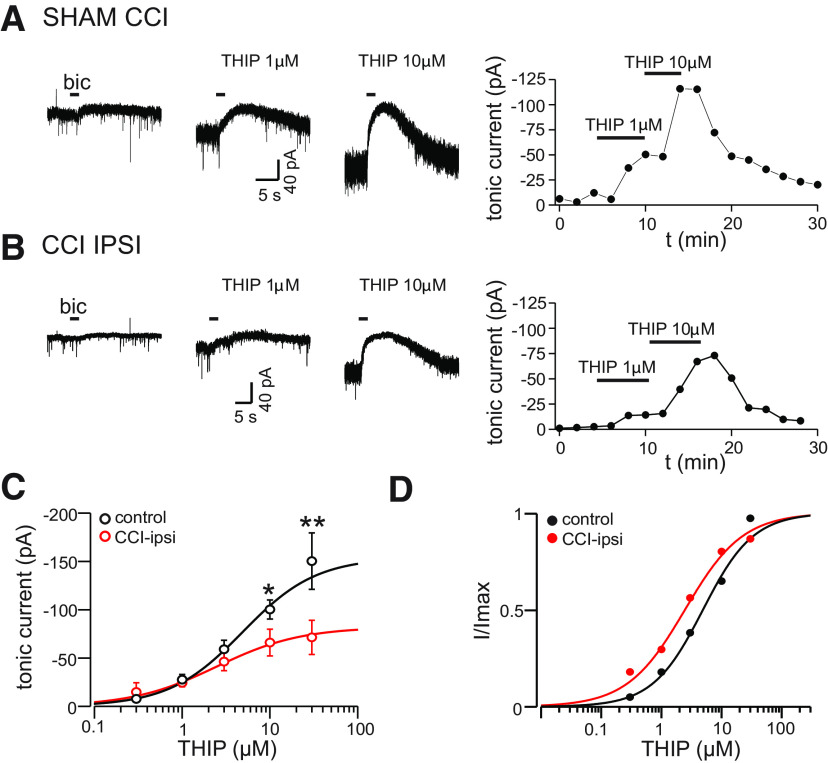
Dose dependence of CCI effects on THIP-induced tonic currents. ***A***, Membrane currents from a sham-treated cell before and during bath application of increasing concentrations of THIP. Right hand panel shows time course of tonic current change for this experiment. ***B***, Membrane currents from a CCI-ipsi cell before and during bath application of THIP, right hand panel shows time course of current change for this experiment. ***C***, Dose–response relationship for THIP-activation of tonic currents in control and CCI-ipsi DGGCs. Data points represent mean ± SEM. CCI reduced current amplitudes with THIP concentrations >3 μm. Solid line is a Hill equation fit to data, the estimated EC_50_ was 4.9 and 2.5 μm for control and CCI-ipsi cells, respectively. ***D***, Normalized dose response data from ***C***. Data and fitted curves were normalized to maximum value (I_max_) for these fits to better illustrate the similar EC_50_ values. Error bars are omitted for clarity. *p < 0.05, **p < 0.01.

We next assessed the concentration dependence for THIP activation of tonic currents for control and CCI-ipsi DGGCs. These data were collected 14–21 d after CCI from two separate groups of CCI-treated animals. Representative recordings of tonic currents from sham and CCI-ipsi cells during bath application of THIP are illustrated in Figure 3*A*,*B*. At concentrations of 10 μm or greater, THIP-induced tonic currents were significantly reduced in CCI-ipsi cells compared with control [10 μm THIP: −100.3 ± 9.9 pA (*n* = 6 cells) vs −66.0 ± 13.8 pA (*n* = 7) for sham/control and CCI-ipsi cells, respectively, *p* < 0.05; 30 μm THIP: −150.3 ± 29.2 pA (*n* = 6 cells) vs −71.4 ± 17.7 pA (*n* = 7) for control and CCI-ipsi cells, respectively, *p* < 0.01]. There was a trend for smaller tonic currents in CCI-ipsi cells with 3 μm THIP, but this was not significant (control: −59.0 ± 9.3 pA vs CCI-ipsi: −46.3 ± 9.4 pA, *n* = 6–8 cells, respectively, *p* = 0.35). The dose–response data were fit with a Hill equation with three degrees of freedom to yield estimated EC_50_ and Hill coefficient of 4.9 and 1.0 μm for control cells and 2.5 and 0.94 μm for CCI-ipsi cells, respectively (Fig. 3*C*). Fit comparison analysis generated an *F* test value of 0.56 (*p* = 0.66), indicating that these fits and associated parameters are not significantly different. These results indicate deficits in THIP-sensitive extrasynaptic GABA_A_ receptors after CCI, an effect that is manifest only at higher concentrations of THIP and is restricted to ipsilateral DGGCs ipsilateral to injury. The similar estimates of THIP affinity for tonic current activation in control and CCI-ipsi DGGCs suggests a pharmacologically-homogenous population of extrasynaptic receptors in both groups.

**Figure 4.**
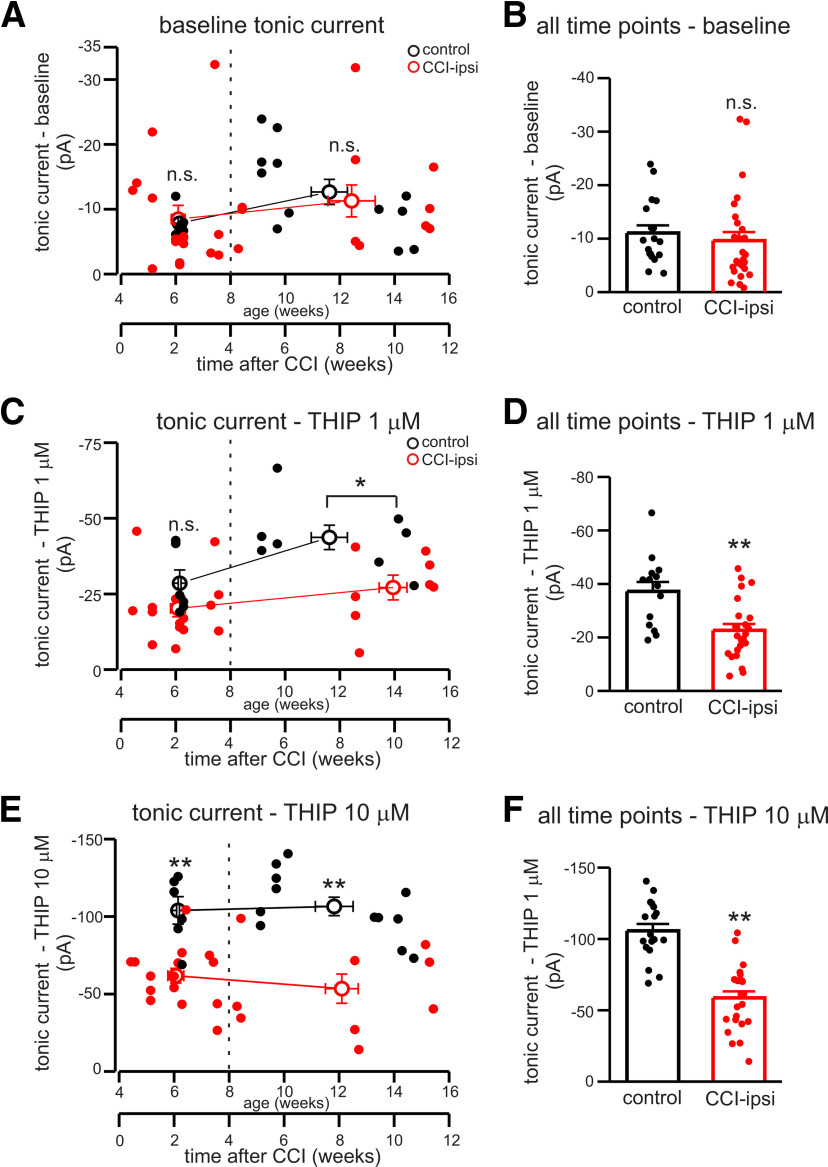
Time dependence of changes in baseline and THIP-induced tonic currents after CCI. ***A***, Baseline tonic current amplitude as a function of age and time after CCI for control and CCI-ipsi cells. Open circles are mean ± SEM of binned data; data binned into groups of cells less than four weeks after CCI or more than four weeks after CCI (dashed line). Solid circles are measurements from individual control (black) or CCI-ipsi (red) cells. ***B***, Mean ± SEM of basal tonic currents at all time points. ***C***, Time course of THIP-induced (1 μm) tonic currents after CCI for CCI-ipsi cells and age-matched controls. ***D***, Mean ± SEM of tonic currents with 1 μm THIP at all time points. Tonic currents with 1 μm THIP were not significantly different compared with control less than four weeks after injury but significant differences were seen in cells more than four weeks after CCI and in ensemble analysis of all time points. ***E***, Time course of tonic currents induced by THIP (10 μm) after CCI for control and CCI-ipsi cells. n.s., not significant; *p < 0.05, **p < 0.01.

Using additional sets of experimental animals, we investigated the time course of changes in tonic currents following CCI. To assess time-dependent changes after CCI, individual tonic current measurements are plotted as a function of age and time after CCI and data were binned into groups less than four weeks or more four weeks after CCI for statistical comparison with age-matched controls (Fig. 4). There were no significant changes in baseline tonic currents after CCI in either group (average time after CCI of 14.6 ± 1.8 or 59.0 ± 6.0 d), or in ensemble analysis of measurements at all time points [two-way ANOVA indicated no effect of CCI (*p* = 0.64) or time (*p* = 0.92); Fig. 4*A*,*B*]. Consistent with data obtained two to three weeks after CCI, THIP-induced (10 μm) tonic currents were significantly reduced in CCI-ipsi cells within the first week of CCI and remained reduced at two months after CCI; ensemble analysis of tonic currents in THIP 10 μm at all time points for these groups of animals had mean values of −105.7 ± 4.8 and −58.7 ± 4.6 pA for control (*n* = 18) and CCI-ipsi (*n* = 24) cells, respectively (two-way ANOVA, *p* < 0.01 for CCI effect, *p* = 0.76 for effect of time after CCI; Fig. 4*E*,*F*). Dose–response data obtained from animals two to three weeks after CCI (Fig. 3*C*) showed a concentration dependence to CCI-related changes in tonic current, with significant differences between control and CCI-ipsi cells seen only at THIP concentrations of 10 μm or greater. However, tonic currents in the presence of 1 μm THIP that were recorded more than four weeks after CCI showed a significant reduction compared with age-matched controls (average time after CCI of 69.6 ± 3.6 d; *p* < 0.01; Fig. 4*C*,*D*). This effect was not seen for data from cells less than four weeks after CCI (average time after CCI of 14.6 ± 1.9 d), but analysis of data at all time points with two-way ANOVA showed a significant reduction of tonic currents in the presence of THIP 1 μm in CCI-ipsi DGGCs (control: −37.2 ± 3.5 pA, *n* = 14; CCI: −22.6 ± 2.4 pA, *n* = 23; *p* < 0.01 for CCI effect). This discrepancy could relate to inter-animal variability, inter-procedural variability in CCI, or time-dependent changes in δ subunit expression after CCI; our data do not suggest significant effect of time after CCI (two-way ANOVA, *p* = 0.28). CCI data presented in Figure 4 was obtained from a total of 26 cells in slices from 16 animals from four separate groups of CCI-treated mice.

These results indicate that alterations in THIP-sensitive, extrasynaptic GABA_A_ receptors occur within one week of injury and persist for at least eight weeks. CCI data presented in Figure 4 was obtained from a total of 26 cells in slices from 16 animals from four separate groups of CCI-treated mice.

### CCI affects synaptic input to DGGCs

**Figure 5.**
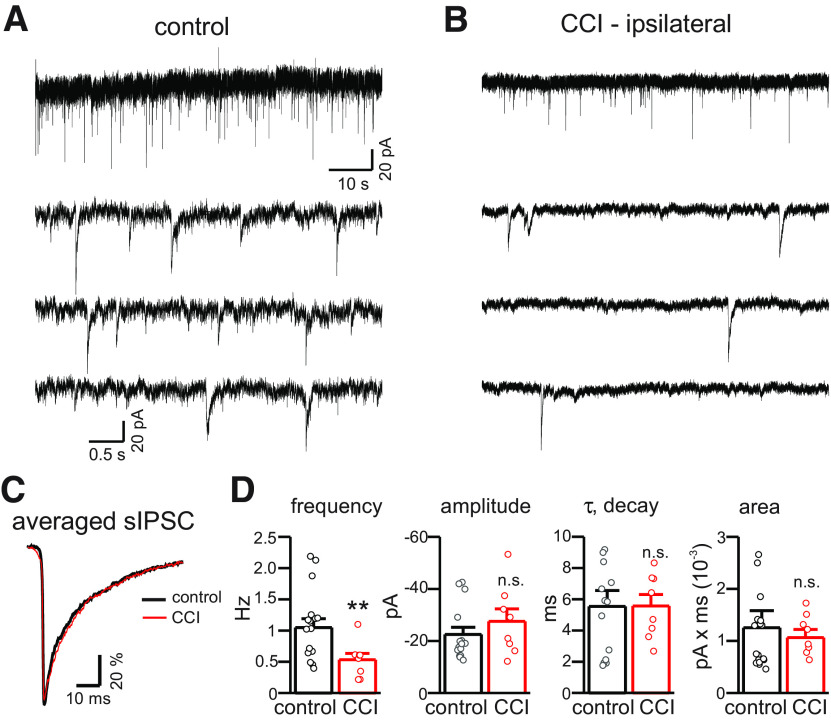
CCI reduces frequency, but not amplitude or kinetics, of sIPSCs in CCI-ipsilateral cells. ***A***, Membrane currents from control and CCI-ipsi DGGCs (2 min of gap-free recording at −70 mV). ***B***, Data from ***A*** on expanded timescale. Reduced frequency of sIPSCs in CCI-ipsi cells is visibly apparent two weeks after CCI. ***C***, Average sIPSC from control and CCI-ipsi DGGCs. Average sIPSCs were normalized to allow comparison of time course and decay kinetics, which were indistinguishable between control and CCI-ipsi cells. ***D***, Mean (±SEM) for sIPSC parameters in control and CCI cells. CCI significantly decreased sIPSC frequency by 59%, but sIPSC amplitude, rise time, decay time constant, and total charge transfer were not significantly affected. Circles represent data from individual cells of control (black) or CCI-treated (red) animals. n.s., not significant. **p < 0.01.

We examined the properties of sIPSCs in control and CCI-ipsi DGGCs (Fig. 5). DGGCs from CCI animals displayed a significant reduction in frequency of sIPSCs compared with control animals (59% reduction on average, control: 0.99 ± 0.1 Hz vs CCI-ipsi: 0.41 ± 0.07 Hz, *n* = 15–18 cells for each condition; one-way ANOVA, *p* < 0.01; Fig. 5*A*,*B*,*D*). However, there were no significant differences of sIPSC amplitude, rise time, or decay time constant in CCI-ipsilateral DGGCs compared with control cells (Fig. 5*C*,*D*). The alteration in sIPSC frequency, but not the properties of sIPSCs themselves, seen after CCI is most consistent with a presynaptic effect because of either a loss of GABAergic interneurons or an alteration of release probability (Hunt et al., 2013; Huusko et al., 2015). Our data does not distinguish between these possibilities nor other potential mechanisms. These results indicate that spontaneous synaptic inhibition of DGGCs is reduced after CCI, consistent with prior studies (Pavlov et al., 2011; Boychuk et al., 2016).

### Immunohistochemical analysis of GABA receptor expression after CCI

**Figure 6.**
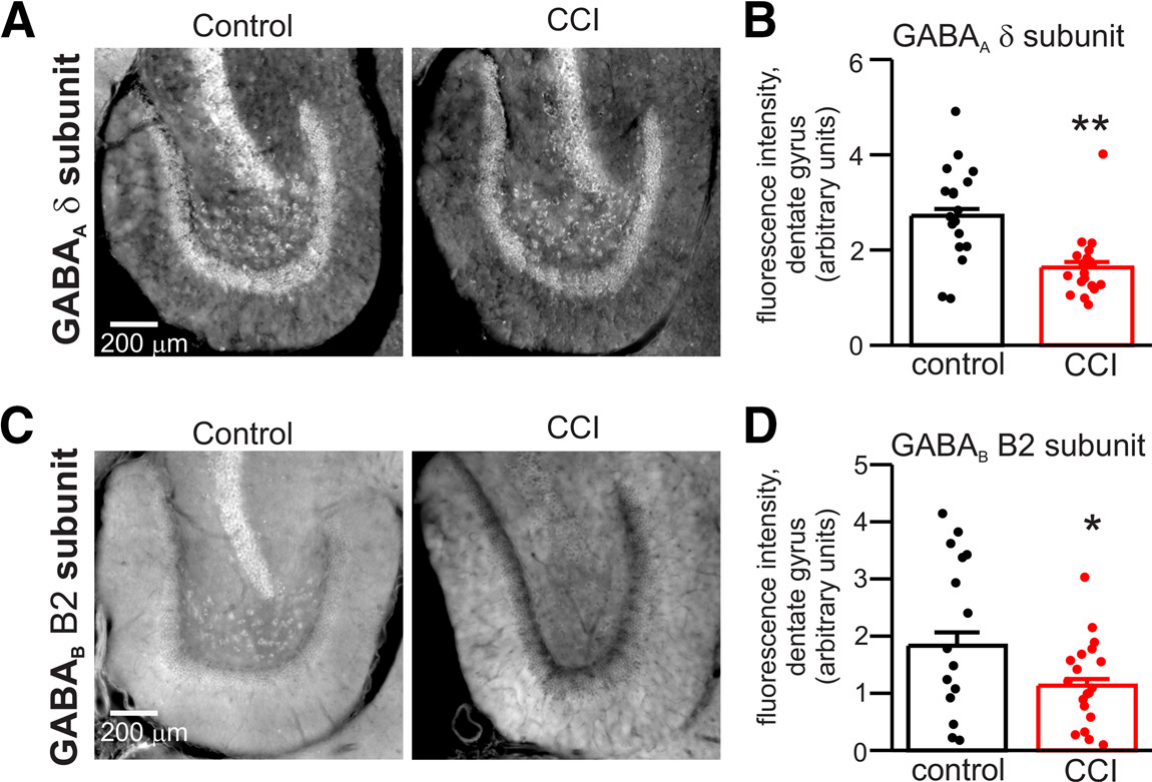
Immunohistochemical analysis of CCI effects on expression of GABA_A_ receptor δ subunits and GABA_B_ receptor B2 subunits in dentate gyrus. ***A***, Immunofluorescent micrographs of staining for GABA_A_ receptor δ subunit in dentate gyrus of control and CCI-ipsi slices. Gray scale images used for quantification are illustrated. ***B***, Mean signal intensity (arbitrary units) for GABA_A_ receptor δ subunit staining of control and CCI-ipsi dentate gyrus. Quantification of signal intensity was normalized to area of ROIs, illustrated data points are the average of area-specific intensity of ROI for entire dentate gyrus (solid circles represent data from individual slices). CCI significantly reduced staining intensity for GABA_A_ receptor δ subunit in dentate gyrus. ***C***, Immunofluorescent micrographs of GABA_B_ receptor B2 subunit staining in dentate gyrus of control and CCI-ipsi slices. ***D***, Mean signal intensity of GABA_B_ receptor B2 subunit staining. CCI significantly reduced intensity of GABA_A_ receptor δ subunit staining in dentate gyrus. *p < 0.005, **p < 0.01.

The reduction of THIP-induced tonic currents after CCI suggests reduced expression of GABA_A_ receptor δ subunits in dentate gyrus after CCI. We assessed expression of GABA_A_ receptor δ subunits by quantifying immunofluorescence in dentate gyrus of control and CCI-treated hippocampus (two weeks after CCI; Schneider Gasser et al., 2006). The IHC confirmed δ subunit expression in cell body layer, ML, and hilar region of dentate gyrus (Fig. 6*A*). Quantification of signal intensity from gray-scale images (normalized to ROI area) showed a significant reduction of staining density for δ subunits in all regions of the dentate gyrus after CCI (GCL, ML, and hilus). On average, CCI caused a 40% reduction in intensity of δ subunit staining across the entire dentate gyrus (*n* = 18–19 slices from three animals under each condition, *p* < 0.01; Fig. 6*B*). δ Subunit staining was reduced in GCL, ML, and hilus by 40%, 39%, and 49%, respectively. The decrease in δ subunit staining is quantitatively similar to the 39–44% reduction in THIP-induced tonic currents aforementioned (i.e., Fig. 4*D*,*F*).

In addition to effects on receptors involved in tonic and phasic inhibition, CCI could also alter signaling via metabotropic GABA_B_ receptors that secondarily affect tonic inhibition (Tao et al., 2013). We investigated the effects of CCI on expression of GABA_B_ receptor B2 subunits in dentate gyrus. Staining for GABA_B_ receptors was present in cell bodies, ML, and hilar region (Fig. 6*C*). Quantification of staining intensity for GABA_B_ B2 subunits showed significant reduction in ipsilateral dentate gyrus after CCI. On average, CCI caused a 38% reduction in staining intensity for GABA_B_ B2 subunits across the entire dentate gyrus (*n* = 15–19 slices from three animals under each condition, *p* < 0.05; Fig. 6*D*). GABA_B_ B2 subunit staining was significantly reduced in GCL and hilus by 58% and 46%, respectively (*p* < 0.05).

Our immunohistochemical results indicating reduced expression of GABA_A_ and GABA_B_ receptor subunits after CCI are consistent with prior studies using western blots and *in situ* hybridization (Raible et al., 2012; Drexel et al., 2015). These supportive results taken together with our electrophysiology data indicate that CCI produces broad dysfunction of GABAergic signaling in DGGCs, with altered function of receptors involved in tonic inhibition, fast and slow synaptic inhibition, and both presynaptic and postsynaptic modulatory actions.

### GABA_B_ receptor modulation of tonic currents is attenuated after CCI

**Figure 7.**
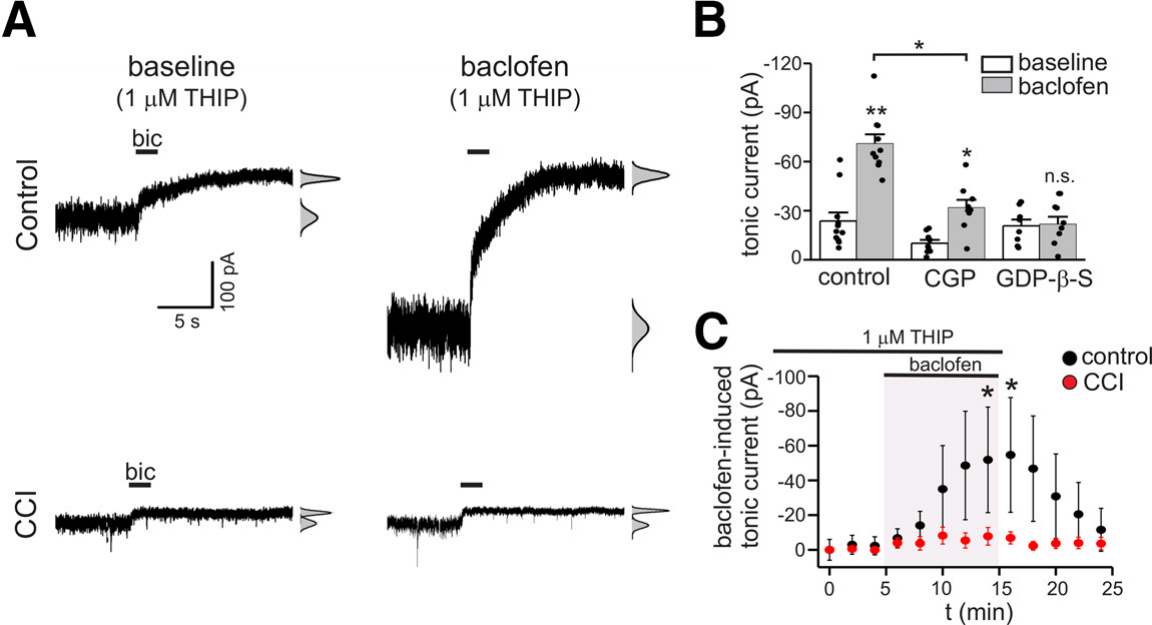
Enhancement of tonic currents by postsynaptic GABA_B_ receptors is attenuated after CCI. ***A***, Membrane currents in response to bicuculline (bic) application in control and CCI-ipsi cells before (left panel traces) and during baclofen (10 μm) application (right-panel traces). All data in this figure were recorded in the presence of low concentrations of THIP (1 μm). ***B***, Mean tonic current at baseline and during baclofen application for cells studied under control conditions, with the GABA_B_ receptor antagonist CGP (10 μm), or with intracellular GDP-β-S (0.5 mm). Baclofen significantly increased tonic currents under control conditions. This effect was partially blocked by CGP, with significant reduction in magnitude of tonic current change compared with control. Disruption of G-protein signaling with intracellular GDP-β-S prevented baclofen-induced tonic current increases. ***C***, Time course of tonic current change during baclofen application (baclofen-induced tonic current) in control and CCI-ipsi cells; n.s., not significant. *p < 0.05, **p < 0.01.

The reduction of GABA_B_ receptor expression after CCI led us to hypothesize that modulation of tonic currents produced by GABA_B_ receptor activation would be attenuated after injury (Tao et al., 2013). To test this, we evaluated the modulation of tonic GABA currents by the GABA_B_ receptor agonist baclofen in control and CCI tissue. These experiments were done in the presence of 1 μm THIP to provide larger, more stable tonic currents while assessing modulation and to constrain our measurements to δ subunit-containing GABA_A_ receptors. In control tissue, tonic currents were increased during bath application of baclofen (BAC, 10 μm), an effect that developed within minutes (baseline: −23.6 ± 5 pA vs BAC: −76.0 ± 33 pA, *n* = 11, *p* = 0.01; Fig. 7*A*,*B*). The GABA_B_ receptor antagonist CGP (10 μm) partially inhibited the response to baclofen, but significantly reduced the change in tonic current induced by baclofen compared with control (tonic current change with BAC: −52.4 ± 28 pA vs tonic current change with CGP + BAC: −8.1 ± 5 pA, *n* = 9–11 cells, *p* < 0.05; Fig. 7*B*). Intracellular solutions containing 0.5 mm GDP-β-S, a GTP analog that inhibits the function of GPCRs (Takahashi et al., 1998; Patenaude et al., 2003; Huang et al., 2015), prevented enhancement of tonic currents during BAC application (Fig. 7*B*). These results indicate that activation of postsynaptic GABA_B_ receptors enhance tonic currents mediated by δ subunit-containing GABA_A_ receptors.

We next examined baclofen modulation of tonic currents in DGGCs two weeks after CCI. Tonic current enhancement by GABA_B_ receptor activation was significantly attenuated in CCI-ipsi DGGCs compared with control (Fig. 7*A*,*C*). On average, THIP-induced tonic currents of CCI-ipsi DGGCs were −15.4 ± 5.8 pA at baseline and increased to −19.6 ± 4.8 pA during baclofen application (*n* = 8, *p* = 0.2; Fig. 7*C*). The increase in tonic current amplitude produced by baclofen in CCI-ipsi DGGCs was, on average, only 8% of that seen in control cells. These findings demonstrate that CCI impairs modulation of tonic inhibition by GABA_B_ receptors.

### GABA_B_ receptor activated K^+^ currents are reduced after CCI

**Figure 8.**
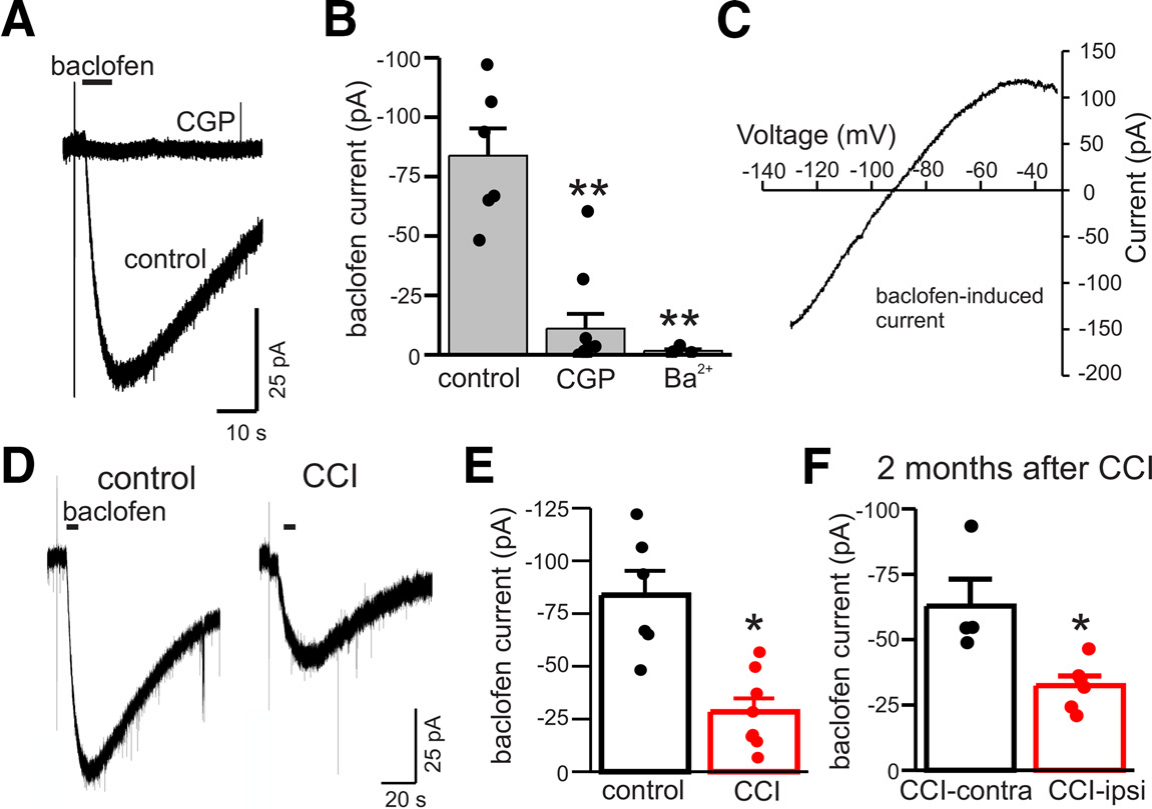
CCI reduces GABA_B_ receptor activated K^+^ currents. ***A***, Membrane currents in response to focal application of baclofen (100 μm, 5-s application) before and during bath application of CGP (10 μm). Currents were measured with K^+^-gluconate pipette solution at −110 mV. ***B***, Mean current induced by baclofen under control conditions, with GGP, or the K^+^ channel blocker Ba^2+^ (100 μm). CGP and Ba^2+^ inhibited baclofen-induced currents by 74% and 82%, respectively. ***C***, Current-voltage plot of ramp current induced by baclofen (subtracted). Baclofen-induced ramp currents were inwardly-rectifying and the illustrated current reversed direction at −90 mV. ***D***, Baclofen-evoked K^+^ currents in control and CCI-ipsi DGGCs. ***E***, Mean GABA_B_ receptor activated K^+^ current in control and CCI-ipsi cells. On average, CCI reduced GABA_B_ receptor-activated K^+^ currents by 66%. ***F***, Mean GABA_B_ receptor activated K^+^ current in CCI-contra and CCI-ipsi DGGCs two months after CCI. GABA_B_ receptor activated K^+^ currents are reduced in CCI-ipsi cells by 48% compared with CCI-contra cells two months after injury. *p < 0.05, **p < 0.01.

Results presented above indicate that CCI reduces GABA_B_ receptor expression and function. To further evaluate functional changes in postsynaptic GABA_B_ receptors after CCI, we directly measured K^+^ currents in response to focal application of baclofen (100 μm). These experiments were done with K^+^-gluconate pipette solution and cells were held at −110 mV during baclofen application to provide sufficient driving force for K^+^ currents. In control DGGCs, baclofen-induced inward currents were sensitive to the GABA_B_ receptor antagonist CGP (10 μm) and the inwardly-rectifying K^+^ channel blocker Ba^2+^ (100 μm; *n* = 5–6 cells for both CGP and Ba^2+^, *p* < 0.01 for both; Fig. 8*A*,*B*). Additionally, we recorded currents in response to ramp voltage commands (from −140 to −30 mV) before and during baclofen application; subtraction of these records yielded the baclofen-induced ramp current. These ramp currents were inwardly-rectifying and had a reversal potential near the equilibrium potential for K^+^ ions (mean reversal potential of −81.2 ± 3 mV, range = −76 to −92 mV, *n* = 4; Fig. 8*C*). These data indicate that focal application of baclofen stimulated GABA_B_ receptor-activated K^+^ channels.

Baclofen-induced K^+^ currents were significantly reduced in CCI-ipsi DGGCs two weeks after CCI. On average, GABA_B_-activated K^+^ currents in CCI-ipsi cells were reduced by 55% compared with control (*n* = 6–8 cells, *p* <0.01) and by 51% compared with the CCI-contra cells (Fig. 8*D*,*E*). To assess whether the changes in GABA_B_ activation of K^+^ currents persisted beyond two weeks after injury a subset of animals was studied two months after CCI. At this later time point, the baclofen-induced K^+^ currents of CCI-ipsi cells were significantly reduced compared with CCI-contra cells (*n* = 4–6, *p* < 0.01; Fig. 8*F*). These data indicate that CCI causes early and persistent functional deficits of GABA_B_ receptors, including activation of postsynaptic K^+^ channels and altered intracellular signaling involved in modulation of tonic GABA currents.

## Discussion

Our results provide evidence for broad disruption of GABAergic signaling in DGGCs in a model of severe TBI (CCI). Our data indicate that CCI produces downregulation of δ subunit-containing GABA_A_ receptors, reduction of spontaneous synaptic input, and functional impairments in GABA_B_ receptor signaling. Immunolabeling of neurons (NEUN) after CCI indicates cell loss in area CA3 with relative preservation of DGGCs targeted for recordings, and suggests that observed physiological changes are not a consequence of simple neurodegeneration and cell death but are a manifestation of surviving neurons. These factors are predicted to dramatically alter inhibitory signaling and hippocampal function with implications for the cognitive and behavioral consequences of TBI, as well as epileptogenesis and recurrent seizures in chronic epilepsy.

### TBI and tonic inhibition

CCI did not affect baseline tonic currents because of endogenous GABA in DGGCs at all time points studied, consistent with prior studies that have measured basal tonic currents one to six months after experimental TBI [CCI or severe fluid percussion injury (FPI); Pavlov et al., 2011; Boychuk et al., 2016; but see Mtchedlishvili et al., 2010; Gupta et al., 2012]. However, tonic currents induced by the δ subunit selective agonist THIP were significantly reduced in DGGCs by one week after CCI and remained reduced up to two months after injury. This effect was restricted to CCI-ipsi cells, suggesting factors that alter properties of DGGCs are limited to the injured hemisphere. At two weeks after CCI, our data identified a concentration dependence to this effect and significant reduction of THIP-induced tonic currents was only seen at concentrations >3 μm. The estimated THIP affinity was similar between control and CCI-ipsi DGGCs (EC_50_ of 4.9 vs 2.5 μm for control and CCI-ipsi, respectively). Our affinity estimates compare well to data on THIP sensitivity of recombinant extrasynaptic GABA_A_ receptors in HEK cells (α4β3δ and α6β3δ), these studies report EC_50_ values ranging from 2.9 to 13 μm (Meera et al., 2011; Mortensen et al., 2010). For comparison, the EC_50_ for THIP activation of synaptic type GABA_A_ receptors containing γ2 subunits is in the range of 69–423 μm and binary receptors composed of only αβ subunits have an intermediate THIP sensitivity. These considerations indicate that the concentrations of THIP we used provided some selectivity for high-affinity extrasynaptic GABA_A_ receptors, which potentially include a mixture of receptors with both αβδ and αβ subunit composition (Mortensen and Smart, 2006; Meera et al., 2011). Thus, CCI causes early and persistent alterations of extrasynaptic GABA_A_ receptor expression and function, a conclusion supported by immunohistochemical analysis showing reduced immunofluorescence of δ subunits after CCI.

Measurements of THIP-induced currents in DGGCs after experimental TBI have provided variable results, with descriptions of either increased THIP-induced currents (Mtchedlishvili et al., 2010; Gupta et al., 2012), decreased THIP-induced currents (Boychuk et al., 2016), or no change in THIP-induced currents (Pavlov et al., 2011). These studies all used THIP concentrations of 1–3 μm; in the light of our data showing a concentration dependence for detecting altered THIP currents after CCI, it is compelling to speculate that use of higher concentrations of THIP may have revealed additional or more consistent alterations of tonic currents after CCI. Technical factors relating to degree of experimental brain injury, timing of measurements after injury, hemisphere under study (ipsilateral vs contralateral), and even factors related to animal strains could account for some of the reported variability. The core finding that CCI reduces THIP currents in ipsilateral DGGCs was observed in six different sets of animals exposed to experimental TBI, and following experimental TBI performed by different investigators; thus, we believe reduced expression of THIP-sensitive extrasynaptic GABA_A_ receptors is a robust and reliable feature of the CCI model.

Irrespective of these variable results, our data raise an important question: if expression of δ subunit-containing extrasynaptic GABA_A_ receptors is reduced after CCI, why are basal, endogenous tonic currents unaltered? This observation suggests that compensatory mechanisms allow DGGCs to maintain physiological levels of tonic inhibition after CCI despite fewer receptors. Boychuk et al. (2016) also observed reduced THIP currents after CCI with paradoxical maintenance of baseline tonic currents that was not because of upregulation of α5 subunit-containing extrasynaptic GABA_A_ receptors or apparent changes in GAT1 function. Estimates of THIP affinity were similar between control and CCI-ipsi DGGCs, arguing against transition to new GABA_A_ receptor subtypes after CCI, but we cannot exclude the emergence of THIP-insensitive, α5 subunit-containing extrasynaptic GABA_A_ receptors (Zhan and Nadler, 2009). Basal tonic inhibition after CCI could also be maintained by elevations of ambient GABA levels (Reeves et al., 1997) or post-translational modifications of extrasynaptic receptors affecting GABA affinity, gating kinetics/open probability, single channel conductance, or even the frequency of spontaneous, GABA-independent channel openings (McCartney et al., 2007; Wlodarczyk et al., 2013). Changes in intracellular Cl^–^ concentration after CCI could also affect basal levels of tonic currents, but this effect would to be minimized by our use of CsCl pipette solutions during whole-cell recordings. Our data do not distinguish between these possibilities, and defining the factors that maintain basal tonic currents after CCI despite downregulation of δ subunit-containing GABA_A_ receptors will require additional study.

### TBI and synaptic inhibition

In contrast to the variable reports of TBI effects on tonic GABA currents, reduced synaptic inhibition in DGGCs is more uniformly described after experimental TBI (Mtchedlishvili et al., 2010; Pavlov et al., 2011; Boychuk et al., 2016). Our analysis of sIPSCs indicates a reduction in sIPSC frequency without changes in amplitude or kinetics after CCI, an effect that was limited to the ipsilateral hemisphere at the time point studied (two weeks after CCI). This pattern of altered phasic inhibition suggests a presynaptic deficit without alteration of postsynaptic GABA_A_ receptor expression or function. Significant loss of GABAergic interneurons is consistently observed in many areas of the brain after TBI, including the amygdala, hippocampus, dentate gyrus, and cortex (Pavlov et al., 2011; Almeida-Suhett et al., 2014; Cantu et al., 2014; Huusko et al., 2015). We suspect that loss of interneurons after CCI contributes to the reduced sIPSC frequency observed, although we did not independently quantify interneuronal loss in our CCI model and other presynaptic and postsynaptic factors may also contribute. Our use of kynurenic acid to block glutamate receptors and isolate GABA_A_ receptor currents is expected to reduce sIPSC frequency and could potentially mask glutamate-dependent, compensatory network activity after CCI (Salin and Prince, 1996; Santhakumar et al., 2001). However, TBI-related changes in both tonic and phasic inhibition have been shown to be independent of glutamate receptor blockade (Pavlov et al., 2011; Gupta et al., 2012).

### GABA_B_ receptor function and TBI

Tonic inhibition is subject to modulation by a variety of receptor subtypes including insulin receptors, dopamine receptors, and GABA_B_ receptors (Jin et al., 2011; Connelly et al., 2013a). The effects of GPCRs (and associated signaling pathways) on tonic inhibition are cell-type specific and depend on the type of extrasynaptic GABA_A_ receptor subunits expressed. For example, tonic inhibition in nigrostriatal D1+ and D2+ medium spiny neurons (MSNs) display distinct and reciprocal patterns of modulation. D2-like receptors (G_i/o_), which decrease PKA activity, reduce tonic currents of D2+ MSNs. In contrast, tonic currents of D1+ MSNs are insensitive to decreased PKA activity, but are increased by D1-like receptors (G_s_) that stimulate PKA activity (Janssen et al., 2009). In hippocampus and thalamus, GABA_B_ receptors (G_i/o_) enhance tonic inhibition in cells expressing GABA_A_ receptor δ subunits (thalamocortical cells, DGGCs, and interneurons), but not in CA1 pyramidal cells (that mainly express α5 subunit-containing receptors) nor δ subunit knock-out mice (Glykys et al., 2008; Connelly et al., 2013b; Tao et al., 2013). Modulation by GPCRs and signaling pathways are mechanisms allowing neurons to dynamically “tune the tone” of extrasynaptic GABA_A_ receptors in response to neuromodulators or ongoing neural activity. These effects will develop and operate over minutes, in contrast to other mechanisms that rapidly (seconds) regulate tonic conductance (e.g., nonvesicular GABA release, voltage-dependent properties of GABA_A_ receptors; Pavlov et al., 2009; Ransom et al., 2010, 2013).

Enhancement of tonic inhibition by GABA_B_ receptors is predicted to reduce the response of DGGCs to excitatory synaptic inputs with consequences for information flow through hippocampus and hippocampal network activity (Pavlov et al., 2009; Connelly et al., 2013b). GABA_B_ receptors are believed to enhance tonic inhibition via increased surface expression of GABA_A_ receptors, including those containing δ and β3 subunits (Kuczewski et al., 2011; Parga and Ransom, 2018). Activation of postsynaptic GABA_B_ receptors, localized to extrasynaptic portions of membrane (Kulik et al., 2003), requires robust patterns of presynaptic activity and GABA spillover (Scanziani, 2000; Lüscher and Slesinger, 2010). It is hypothesized that periodic increases in extracellular GABA during pathophysiological neural activity, such as interictal bursting and seizures (Perreault et al., 1992; During and Spencer, 1993; Markwardt et al., 2009), could activate postsynaptic GABA_B_ receptors to provide adaptive, feed-forward increases in tonic inhibition. Our data indicate that modulation of tonic inhibition by GABA_B_ receptors is markedly attenuated after CCI. The enhancement of tonic inhibition by GABA_B_ receptors (mediated by G_i/o_ signaling) could be occluded after TBI when cAMP levels and PKA activity are reduced in hippocampus (Atkins et al., 2007; Titus et al., 2013a). However, TBI-associated changes in cAMP/PKA activity recover within days of injury so are not expected to contribute to the deficits in GABA_B_ modulation of tonic currents we observed two weeks after injury. Moreover, our immunohistochemical analysis and direct measurements of GABA_B_ receptor-activated K^+^ currents demonstrate reduced expression and function of GABA_B_ receptors after CCI. This downregulation explains, at least in part, the impaired modulation of tonic currents after CCI. It remains to be shown whether synaptic GABA_B_ responses/slow IPSCs are also affected by CCI, but altered GABA_B_ expression and GIRK channel function is predicted to significantly impact behavior of DGGCs independent of modulatory actions on tonic inhibition (Lüscher and Slesinger, 2010). To the best of our knowledge, this study is the first description of functional deficits of GABA_B_ receptors after experimental TBI (but see Drexel et al., 2015).

### Functional implications for altered GABAergic signaling after TBI

Our findings in the CCI model are most relevant to severe TBI, defined by prolonged (>24 h) loss of consciousness or structural brain abnormalities/hemorrhage, and severe TBI confers a high risk of developing epilepsy (second only to subarachnoid hemorrhage as risk factor for acquired epilepsy; Annegers et al., 1998; Garga and Lowenstein, 2006). In addition to deficits related to neuroanatomical injuries and seizures, sequelae of severe TBI frequently includes cognitive dysfunction and mood disorders (Morissette et al., 2011; Paterno et al., 2017). Numerous cellular and molecular processes contribute to the complex pathophysiology and outcome of TBI, including neuronal death (Huusko and Pitkänen, 2014), NMDA receptor function/CAMKII expression (Schwarzbach et al., 2006), mossy fiber sprouting (Hunt et al., 2009), intracellular signaling pathways (Yang et al., 1993; Kobori et al., 2014), immune/inflammatory mechanisms (Schwarzmaier and Plesnila, 2014), and alterations in GABAergic signaling (Hunt et al., 2013). The persistent effects of CCI on expression and regulation of δ subunit-containing extrasynaptic GABA_A_ receptors described here are of particular interest, because tonic inhibition has established effects on many of the physiological and pathophysiological processes affected by TBI, including learning and memory (Cheng et al., 2006), anxiety-related and mood-related behaviors (Smith et al., 2007), and epileptic seizures (Chuang and Reddy, 2018).

Functional deficits in δ subunit-containing GABA_A_ receptors and GABA_B_ receptors after CCI are predicted to alter cellular/network activity and behavior. Furthermore, the consequences of these functional deficits are predicted to be greatest during robust neural activity accompanied by transient elevations of GABA concentration. Because only a fraction of extrasynaptic GABA_A_ receptors are active under basal conditions, activity-dependent increases in [GABA] will activate additional, unbound receptors and increase tonic inhibition (Wei et al., 2003; Ransom et al., 2013). Reduced expression of both GABA_A_ and GABA_B_ receptors after CCI will impair the ability of DGGCs to rapidly adjust inhibitory tone in response to ongoing neural activity. These impairments will affect the integration and processing of inputs arriving from cortex, recurrent mossy fibers, and local interneurons and promote hyperexcitability. Although our data are limited to DGGCs, the changes in GABAergic signaling caused by CCI are expected to contribute to broader hippocampal dysfunction and the clinical consequences of TBI, including alterations in synaptic plasticity that can impact memory and epileptogenesis (Titus et al., 2013b; Wu et al., 2014). GABA_A_ receptor δ subunits are also an emerging therapeutic target for mood disorders, and neurosteroid analogs that potentiate tonic inhibition (brexanolone, SAGE-217) are effective treatments for major depression and postpartum depression (Kanes et al., 2017; Gunduz-Bruce et al., 2019). The development of mood disorder after TBI likely involves more widespread networks than just the hippocampus, and *in situ* hybridization experiments indicating reduced expression of δ subunits in cortex and thalamus after experimental TBI support a possible role of δ subunit in post-TBI depression (Drexel et al., 2015).

Defining the cellular and molecular changes triggered by TBI and their relative contribution to behavioral and cognitive outcomes and epileptogenesis/seizures is a daunting challenge but is essential to develop novel, effective strategies and interventions to improve outcomes. Of the myriad effects of TBI, those that occur reliably and persist beyond the acute phase deserve the greatest attention and represent opportunity for therapeutic intervention. Our data and prior work compel us to conclude that reduced expression of extrasynaptic GABA_A_ receptors and GABA_B_ receptors are reliable and persistent consequences of TBI. Future work defining the proximal factors that generate persistent functional changes after TBI could inform strategies to limit the cellular and molecular changes that underlie cognitive dysfunction and the development of epilepsy.

## Synthesis

Reviewing Editor: Katalin Toth, University of Ottawa Decisions are customarily a result of the Reviewing Editor and the peer reviewers coming together and discussing their recommendations until a consensus is reached. When revisions are invited, a fact-based synthesis statement explaining their decision and outlining what is needed to prepare a revision will be listed below. The following reviewer(s) agreed to reveal their identity: Robert Bonin.

The authors have significantly improved this review article. Both reviewers were very positive about the effort the authors made with this revision. However, there are few areas that still need further improvement:

1. The authors should provide details about the ANOVA tests they used.

2. The authors claim that they observed selective cell loss in the CA2, but their figure shows cell loss in the CA3 area, the various areas don’t seem to be correctly labelled on Figure 1. This figure should be corrected for the labels and the images should not crop of the dentate gyrus. If the authors aim to show selective cell loss in the CA2, that would require further experiments to identify this region.

3. Figure 5 shows changes in spontaneous IPSCs, however the authors used CsCl filled electrodes and they recorded at -70mV. Under these conditions both EPSCs and IPSCs would be observed. It is unclear how the authors differentiated between EPSCs and IPSCs. Did they block the EPSCs? this information is missing from the manuscript and should be added.

Minor: On Page 13, line 266 It should be Fig. 6B instead of Fig. 5B

Rev. #1.

The methods indicate that ANOVA followed by pairwise comparisons was used. However, it is unclear whet types of ANOVA was used. The design includes effects of injury and drug treatment however there is no mention of two-way ANOVA. There is also effect of age considered which may need mixed design ANOVA and dose response curves which may need repeated measures or mixed design ANOVA.

The authors have addressed several concerns with additional data and explanations. The CCI mediated changes in THIP and GABAB sensitive tonic GABA currents as well as changes in GABAb currents and sIPSCs are findings that are of definite merit. However, some concerns and inconsistencies remain

1. The qualitative validation of the CCI model is inadequate and inaccurate. The authors claim CA2 cell loss without specific staining for CA2. The image suggests extensive CA3 loss. Since the focus of the physiological analysis is the dentate gyrus, the lack of emphasis of dentate hilar neuropathology is of concern. Illustrative sections don’t appear level matched, parts of the dentate are cut off and lacks scale bar as suggested in the legend.

2. Statistics: Was Two-way ANOVA use to test effect of injury and drug? Or was One-way ANOVA performed on sham vs. CCI-ipsi vs. CCI-contra? More information on statistical outcomes and tests used needs to be included.

3. Fig. 6 lacks scale bars

Rev. #2.

The authors have done a commendable job with the revisions and their detailed response is appreciated. I have no further comments.
